# Identification and characterization of Cardiac Glycosides as senolytic compounds

**DOI:** 10.1038/s41467-019-12888-x

**Published:** 2019-10-21

**Authors:** Francisco Triana-Martínez, Pilar Picallos-Rabina, Sabela Da Silva-Álvarez, Federico Pietrocola, Susana Llanos, Verónica Rodilla, Enrica Soprano, Pablo Pedrosa, Alba Ferreirós, Marta Barradas, Fernanda Hernández-González, Marta Lalinde, Neus Prats, Cristina Bernadó, Patricia González, María Gómez, Maria P. Ikonomopoulou, Pablo J. Fernández-Marcos, Tomás García-Caballero, Pablo del Pino, Joaquín Arribas, Anxo Vidal, Miguel González-Barcia, Manuel Serrano, María I. Loza, Eduardo Domínguez, Manuel Collado

**Affiliations:** 1 Laboratory of Stem Cells in Cancer and Aging, Health Research Institute of Santiago de Compostela (IDIS), Xerencia de Xestión Integrada de Santiago (XXIS/SERGAS), E15706 Santiago de Compostela, Spain; 2 BioFarma, Center for Research in Molecular Medicine and Chronic Diseases (CIMUS), Universidade de Santiago de Compostela, Health Research Institute of Santiago de Compostela (IDIS), Santiago de Compostela, Spain; 3 Institute for Research in Biomedicine (IRB Barcelona), The Barcelona Institute of Science and Technology (BIST), 08028 Barcelona, Spain; 4 DNA Replication Group, Spanish National Cancer Research Centre (CNIO), 28029 Madrid, Spain; 5 Preclinical Research Program, Vall d´Hebron Institute of Oncology (VHIO) and CIBERONC, Barcelona, Spain; 6 Centro Singular de Investigación en Química Biolóxica e Materiais Moleculares (CiQUS) and Departamento de Física de Partículas, Universidade de Santiago de Compostela, 15782 Santiago de Compostela, Spain; 7 Metabolic Syndrome Group, Madrid Institute for Advanced Studies (IMDEA) in Food, CEI UAM+CSIC, Madrid, E28049 Spain; 8 Department of Pulmonology, ICR, Hospital Clinic, Instituto de Investigaciones Biomedicas August Pi i Sunyer (IDIBAPS), Barcelona, 08036 Spain; 9 Histopathology Unit, Spanish National Cancer Research Centre (CNIO), 28029 Madrid, Spain; 10 Translational Venomics Group, Madrid Institute for Advanced Studies (IMDEA) in Food, CEI UAM+CSIC, Madrid, E28049 Spain; 11 Departamento de Ciencias Morfológicas, Facultad de Medicina. USC. Xerencia de Xestión Integrada de Santiago (XXIS/SERGAS), E15706 Santiago de Compostela, Spain; 12 Catalan Institution for Research and Advanced Studies (ICREA), 08010 Barcelona, Spain; 13 CiCLOn, Centro Singular de Investigación en Medicina Molecular y Enfermedades Crónicas (CIMUS), Universidade de Santiago de Compostela, Instituto de Investigación Sanitaria de Santiago de Compostela (IDIS), E15782 Santiago de Compostela, Spain; 14 Servicio de Farmacia, Xerencia de Xestión Integrada de Santiago (XXIS/SERGAS), E15706 Santiago de Compostela, Spain

**Keywords:** Senescence, Drug development

## Abstract

Compounds with specific cytotoxic activity in senescent cells, or senolytics, support the causal involvement of senescence in aging and offer therapeutic interventions. Here we report the identification of Cardiac Glycosides (CGs) as a family of compounds with senolytic activity. CGs, by targeting the Na+/K+ATPase pump, cause a disbalanced electrochemical gradient within the cell causing depolarization and acidification. Senescent cells present a slightly depolarized plasma membrane and higher concentrations of H+, making them more susceptible to the action of CGs. These vulnerabilities can be exploited for therapeutic purposes as evidenced by the in vivo eradication of tumors xenografted in mice after treatment with the combination of a senogenic and a senolytic drug. The senolytic effect of CGs is also effective in the elimination of senescence-induced lung fibrosis. This experimental approach allows the identification of compounds with senolytic activity that could potentially be used to develop effective treatments against age-related diseases.

## Abstract (web summary)

Senolytic compounds have the ability to eliminate senescent cells from tissues and have been shown to be beneficial in various animal models of age-related diseases. Here the authors show that cardiac glycosides commonly used for heart diseases have senolytic properties in humanized mouse models of tumorigenesis and lung fibrosis.

## Introduction

Senescence is a crucial cellular response acting as a morphogenetic force during embryogenesis, contributing to tissue remodeling and homeostasis in the adult, and protecting against cancer development^1^. Senescent cells are characterized by a stable cell cycle arrest that is relevant for its tumor suppressive function and by secreting a complex mixture of soluble factors with immunomodulatory, extracellular matrix remodeling and proliferative activity collectively known as the SASP (senescence-associated secretory phenotype)^2^. This SASP seems to act during development contributing to embryo morphogenesis, and to provide signals for immune clearance of senescence cells and to promote repair in adult damaged tissue^3–5^. Excessive production of senescent cells due to chronic inducing signals or a failure to remove them efficiently can lead to the accumulation of damaged cells with impaired proliferative capacity and emitting signals with negative consequences^1^. Experimental evidence points to this accumulation of senescent cells with time as a cause of aging^6,7^. Senescent cells are prominent in tissues from old individuals and in patients of premature aging syndromes, at sites of age-related disease^8^. This build-up of senescent cells in aged tissue could impair tissue function by the incapacity to proliferate of the senescent cells and by the detrimental effect of SASP factors on neighboring cells^9^. Using genetically modified mouse models to allow the elimination of cells with induced expression of p16 (the product of gene *Cdkn2a*), one of the main factors in senescence induction, supports this notion^10,11^. Removal of senescence cells improves the healthspan of progeroid and old mice.

Oncogene induction in vitro and in vivo triggers a senescence response that limits the proliferation of damaged cells effectively preventing tumor development^12^. This powerful tumor suppressive function of cell senescence has been envisioned as a promising alternative to the use of cytotoxic drugs^13^. In fact, evidence in mouse models of cancer and from archived patient samples shows that senescence induction contributes to treatment outcome^14–16^. However, the persistence of senescent tumor cells secreting SASP factor with potentially lethal activity represents a challenge for the development of a pro-senescence therapy of cancer. Senescent tumor cells have been suggested to promote relapse and metastatic tumor growth^17,18^. In addition, chemo and radiotherapy-induced senescence of normal healthy tissue could underlie the adverse effects often associated with antitumor drugs^19^ and radiation^20–22^.

Apart from the characteristic growth arrest and the SASP, senescent cells induce resistance to cell death^23,24^. Contrary to apoptosis, the senescence response generates damaged cells that can remain in tissues for a prolonged period of time. Recently, several compounds have been identified with selective cytotoxic activity on senescent cells^25–28^. This so-called senolytic drugs have the potential to remove specifically senescent cells in vitro and in vivo, demonstrating a beneficial therapeutic effect against aging and age-related diseases. Identifying the mechanisms of apoptosis resistance in senescence cells might be helpful to develop novel senolytic drugs. Alternatively, high-throughput screening of chemical libraries might allow the unbiased identification of senolytic drugs whose action will rely on unidentified vulnerabilities of senescent cells^29^.

With this later idea in mind, we develop a cell-based screening strategy to identify senolytic compounds that we test using a chemical repositioning library. Using this system, we identify Cardiac Glycosides as a family of compounds showing senolytic activity and demonstrate its potential use in combination with senogenic drugs as a potent antitumor strategy. In addition, we describe the molecular basis for this senolytic effect.

## Results

### High-throughput compound screening for senolytic activity

**Fig. 1:**
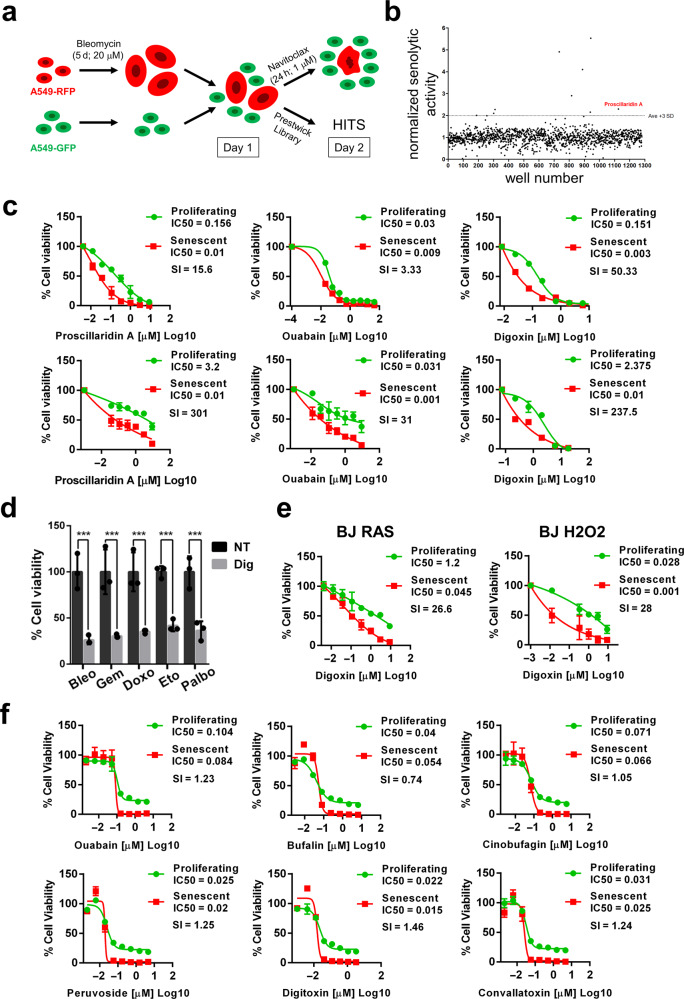
High-throughput screenings of senolytic compounds. **a** Schematic diagram of the experimental system used to screen compounds for potential senolytic activity. **b** Scatter plot representing the normalized senolytic activity obtained with each compound present in the Prestwick Chemical Library. Proscillaridin A value highlighted in red. **c** IC50 curves obtained with increasing concentrations of Proscillaridin A, Ouabain and Digoxin (from left to right) in A549 tumor cells (upper panels) and BJ primary fibroblasts (bottom panels). Actual IC50 values and Senolytic Indexes (SI) are also shown for each condition. **d** Relative cell viability (%) after Digoxin or vehicle treatment of A549 cells rendered senescent by different chemotherapeutic reagents (Bleo: Bleomycin; Gem: Gemcitabine; Doxo: Doxorubicin; Eto: Etoposide; and Palbo: Palbociclib). *n* = 3 independent experiments. Statistical significance was assessed by the two-tailed Student's t-test: ***p < 0.001. **e** IC50 curves of BJ primary fibroblasts induced to senescence by oncogenic RAS overexpression (left panel) or H2O2 treatment (right panel) treated with increasing concentrations of Digoxin. **f** IC50 curves of SK-MEL-103 melanoma cells induced to senescence by Palbociclib and treated with increasing concentrations of the indicated CGs identified on a screening for senolytic activities present in compounds obtained from plant extracts. Data correspond to the average ± s.d. Source data for these experiments are provided as a Source Data file

We set up the conditions to screen for senolytic compounds using as target cells a human lung adenocarcinoma cell line, A549 (Fig. 1a). As a proof-of-concept, we screened for compounds showing senolytic activity in the Prestwick chemical library, a collection of 1280 small molecules, mostly FDA/EMA approved drugs, ideal for pharmacological repositioning. We derived clonal GFP and RFP expressing versions of A549 by lentiviral transduction and antibiotic selection, and induced senescence by treatment with the chemotherapeutic drug Bleomycin (20 μM; 5 days), as previously described^30^. We plated mixed cultures of proliferating A549-GFP and senescent A549-RFP cells (in a 1:3 proportion) in 384-well plates. One day later, we counted the number of GFP and RFP cells using Operetta High-Content Imaging System, added the compounds in the library (10 μM) and repeated the counts 24 h later. For each plate we included untreated wells as negative control or the well-known senolytic drug, Navitoclax^22^ (1 μM) as positive control to calculate its individual *Z*′ score. We calculated the percentage of change in the cell populations before and after the treatments and obtained a ratio that was used to select for potential hits. A total of 9 compounds showed the highest activity with a ratio of change in the cell population above the threshold (average for the whole population plus 3 standard deviations) (Fig. 1b). These initial hits were further subjected to validation at two concentrations (10 and 1 μM) and were confronted with the results produced by our positive control, Navitoclax. One of the compounds that passed this confirmatory step was Proscillaridin A.

Proscillaridin A belongs to the Cardiac Glycosides (GCs) family of compounds, derived from the foxglove plant (*Digitalis purpurea*) whose therapeutic benefits were originally described more than two centuries ago^31^, and that despite their toxicity are used in the clinic for the treatment of congestive heart failure and cardiac arrhythmias^32^. However, Proscillaridin A is not currently used in the clinic and Digoxin is the most widely used CG for the management of heart failure and in chronic atrial fibrillation^33^. We decided to confront Proscillaridin A, Ouabain, one of the most studied CGs, and Digoxin for their relative senolytic activity. Using A549 tumor cells and primary human BJ fibroblasts induced to senescence by Bleomycin treatment we confirmed that all of them showed senolytic activity, with IC50 values in the nanomolar range and with senolytic indexes ranging from 3.33 (Ouabain in A549 cells) to 301 (Proscillaridin in BJ cells) (Fig. 1c). The cell killing activity of CGs was specific of the senescence state, since quiescent cells showed lower sensitivity than senescent cells (Supplementary Fig. 1a). Other tumor and primary cell lines confirmed the universality of Digoxin senolytic activity (Supplementary Fig. 1b, c). Furthermore, primary chondrocytes from healthy and osteoarthritic donors were also tested for Digoxin senolytic activity. We confirmed that osteoarthritic chondrocytes are positive for senescence-associated beta-galactosidase (SABG) and validated that Digoxin treatment reduced their viability compared to cells from healthy individuals (Supplementary Fig. 1d, e). In contrast, Digoxin did not show any senolytic activity on mouse embryo fibroblasts due to the well-known insensitivity of mouse cells to CGs (Supplementary Fig. 1f)^34^. This senolytic effect was not observed exclusively in cells induced to senescence by treatment with Bleomycin. Using other senescence-inducing chemotherapeutic drugs, Gemcitabine, Doxorubicine, Etoposide and Palbociclib, we observed that in all cases Digoxin showed a similar senolytic effect (Fig. 1d). To test whether Digoxin had senolytic activity on cells induced to senescence by other senescence-inducing stimuli, we overexpressed an oncogenic version of the *HRAS* gene on human primary BJ fibroblasts or treated these same cells with H_2_O_2_. In both cases, Digoxin treatment led to the preferential killing of the senescent cells (Fig. 1e).

In an independent manner, we set up another screening using a melanoma cell line, SK-MEL-103, induced to senescence by Palbociclib treatment (5 μM; 7 days). This time, we screened 480 compounds from the GPNCL library of natural compounds (Greenpharma Natural Chemical Library) and 502 compounds from the SCREEN-WELL^®^ Natural Product library (ENZO). Interestingly, all the six compounds that were found to be positive in this screening belong to the CG family, namely Ouabain, Bufalin, Cinobufagin, Peruvocide, Digitoxin, and Convallatoxin (Fig. 1f). These compounds represent different sub-families within the Cardiac Glycoside family, supporting the idea that the observed senolytic activity is a general feature of CGs. Finally, using this same cell line and a library of 200 venoms and venom-derived peptides and compounds from snakes, spiders, toads, bees, centipedes, ants, octopus and lizards, we identified a group of venoms showing senolytic activity, all of them derived from toads (Supplementary Fig. 1g). Most of the species that were positive for senolytic activity are well-known for containing Bufadienolides, a subgroup of CGs^35,36^.

In summary, we have identified in three independent high-throughput screenings several compounds from the CG family with specific cytotoxic activity against senescent human primary and cancer cells independently of the method used to induce senescence.

### Cardiac Glycosides kill senescent cells by apoptosis

**Fig. 2:**
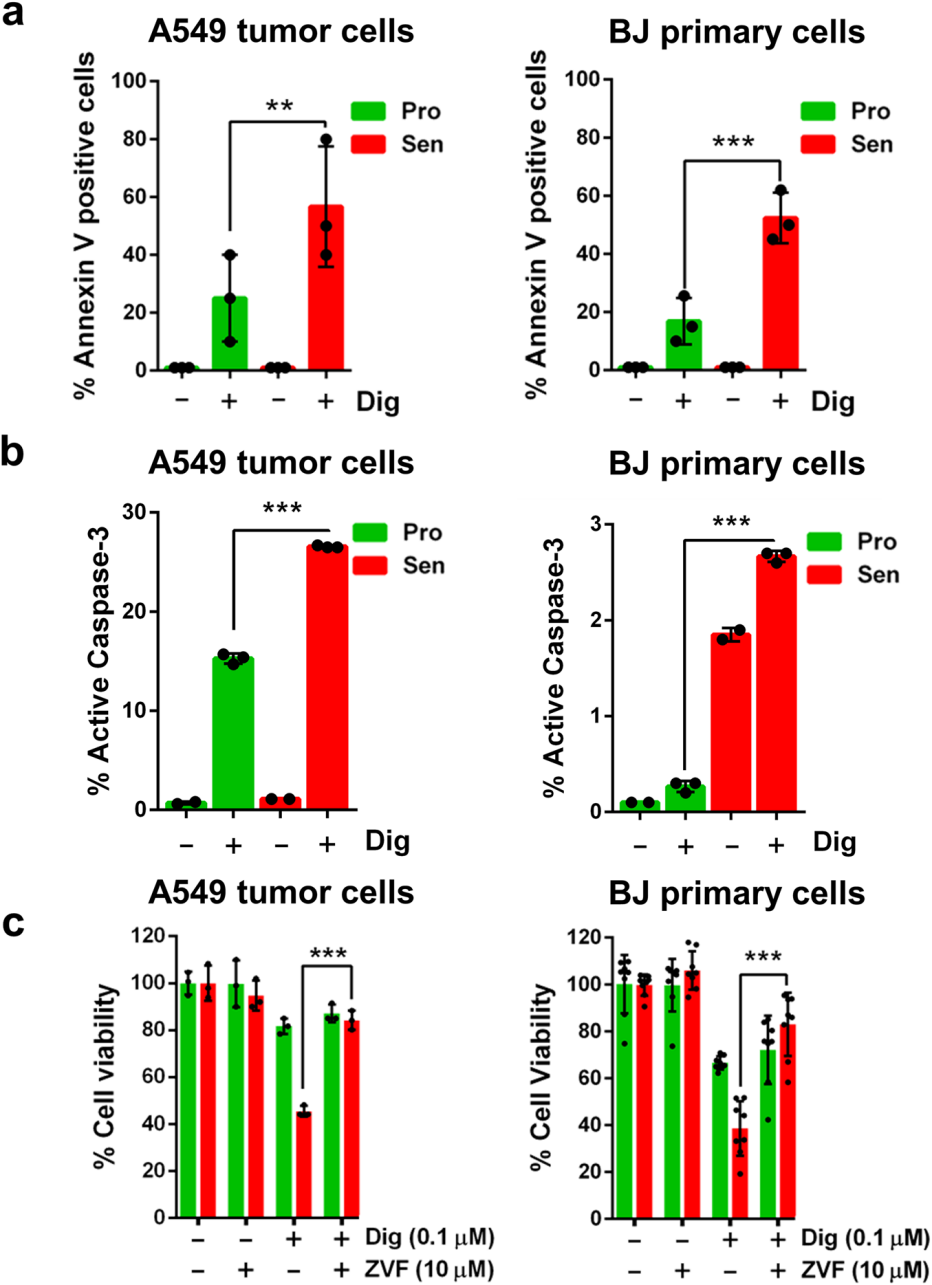
CGs kill senescent cells by inducing apoptosis. **a** Annexin V positive cells (%) in proliferating (Pro, in green) and senescence (Sen, in red) A549 cells (left panel) or primary BJ fibroblasts (right panel) after Digoxin treatment. **b** Active Caspase-3 positive cells (%) in proliferating (Pro, in green) and senescence (Sen, in red) A549 cells (left panel) or primary BJ fibroblasts (right panel) after Digoxin treatment. **c** Relative cell viability (%) of proliferative (Pro) or senescence (Sen) A549 cells treated with pan caspase inhibitor Z-VAD-FMK (ZVF), Digoxin (Dig) or the combination, as indicated. *n* = 3 independent experiments. All data correspond to the average ± s.d. Statistical significance was assessed by the two-tailed Student's t-test: ***p < 0.001; **p < 0.01. Source data for these experiments are provided as a Source Data file

To investigate the mechanism by which CGs were inducing cell death in senescent cells we checked apoptosis induction. First, we analyzed Annexin V staining as a surrogate marker of apoptotic cell death using an Annexin V-FITC staining kit and flow cytometry analysis. Digoxin-induced senolysis clearly increased the percentage of Annexin V positive A549 or BJ cells induced to senescence by Bleomycin treatment (Fig. 2a). Similarly, we also observed induction of active Caspase-3, another marker of apoptosis (Fig. 2b). Finally, treatment with Z-VAD-FMK, an irreversible pan caspase inhibitor, to block apoptosis resulted in protection from the cell death induced by Digoxin (Fig. 2c). In contrast, when we used inhibitors of other cell death pathways such as ferroptosis and necroptosis we did not observe any protection Supplementary Fig. 2). Thus, Digoxin provokes cell death mainly by inducing apoptosis.

### The Na+/K+ATPase is the senolytic target of CGs

**Fig. 3:**
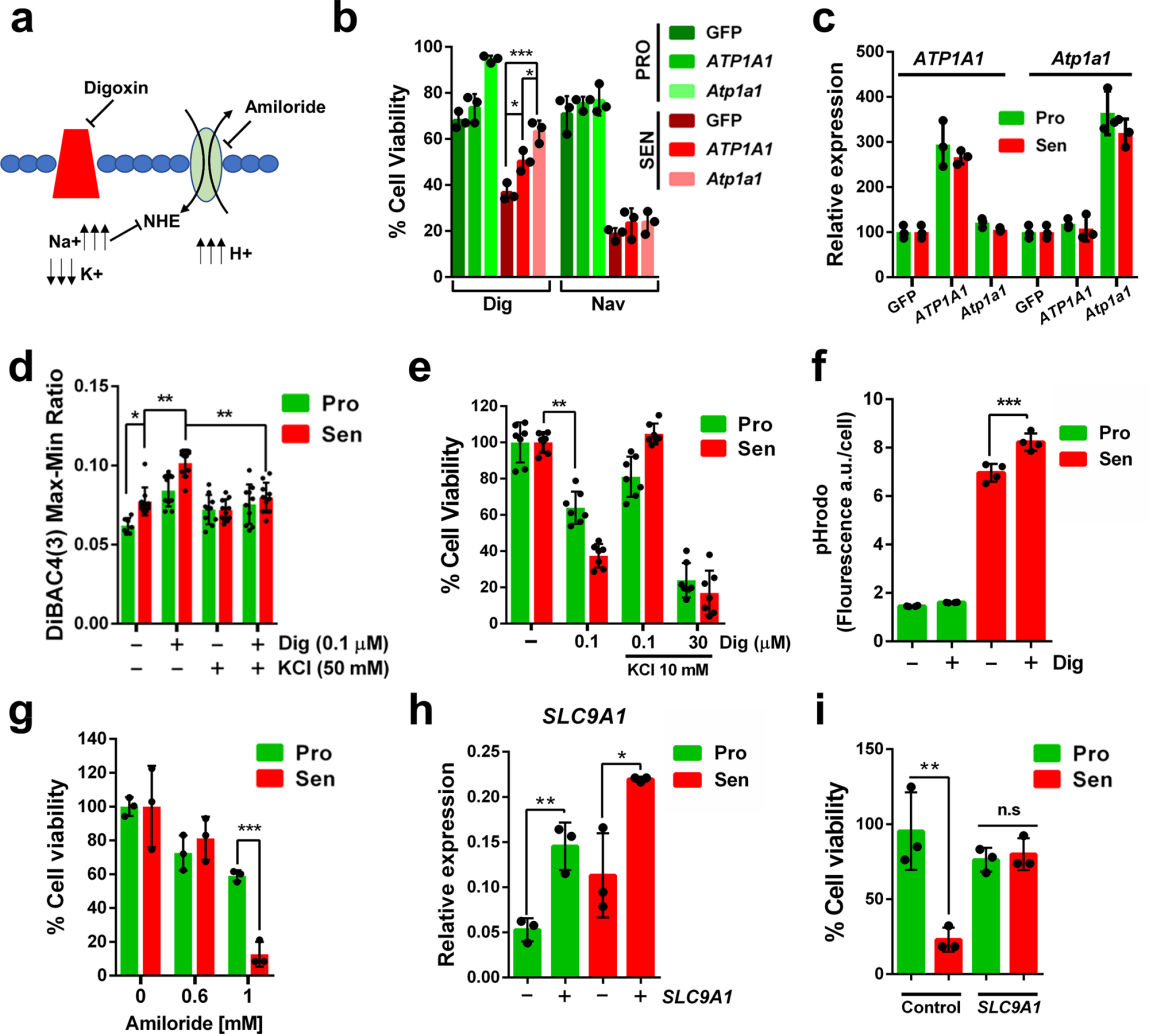
Mechanism of action of Digoxin-induced senolysis. **a** Diagram showing the activity of Digoxin. **b** Relative cell viability (%) of proliferative (green) or senescence (red) A549 cells overexpressing human or mouse *ATP1A1* or *Atp1a1*, respectively, and treated with Digoxin or Navitoclax. Statistical analysis with Anova with Tuckey test: ***p < 0.001; *p < 0.05; **c** and mRNA expression (relative to *GAPDH*) of *ATP1A1* (left) and *Atp1a1* (right). *n* = 3 independent experiments for both **d** Membrane potential determination of proliferative (green) or senescence (red) A549 cells treated with Digoxin and KCl, using fluorescent probe DiBAC4(3). *n* = 10 biologically independent samples. Statistical significance by two-tailed Student's t-test: **p < 0.01; *p < 0.05. **e** Relative cell viability (%) of proliferative (green) or senescence (red) A549 cells treated with Digoxin and KCl. *n* = 7 biologically independent samples. Statistical analysis with Anova with Tuckey test: **p < 0.01. **f** Determination of the relative intracellular H+concentration in proliferative (green) or senescence (red) A549 cells treated or not with Digoxin. *n* = 4 biologically independent samples. Statistical significance by two-tailed Student's t-test: ***p < 0.001. **g** Relative cell viability (%) of proliferative (green) or senescence (red) A549 cells treated or not with Amiloride. *n* = 3 biologically independent samples. Statistical significance by two-tailed Student's t-test: ***p < 0.001. **h** Relative mRNA expression of *SLC9A1* in proliferative (green) or senescence (red) A549 cells overexpressing or not *SLC9A1* as indicated. *n* = 3 biologically independent samples. Statistical significance by two-tailed Student's t-test: **p < 0.01; *p *<* 0.05. **i** Relative cell viability (%) of proliferative (green) or senescence (red) A549 cells treated with Digoxin and exogenously expressing *SLC9A1* or GFP. *n* = 3 independent experiments. Statistical significance by two-tailed Student's t-test: **p < 0.01; n.s. not significant. All data correspond to the average ± s.d. Source data for these experiments are provided as a Source Data file

CGs mechanism of action consists mainly in the inhibition of the Na+/K+ATPase pump, a plasma membrane enzyme that pumps sodium out of the cell while pumping potassium into the cell against their respective concentration gradients (Fig. 3a)^37,38^. The inhibitory action is produced through binding to the alpha subunits of the Na+/K+ATPase pump, the product of the *ATP1A1*, *ATP1A2*, *ATP1A3* and *ATP1A4* genes^34^. We reasoned that overexpression of the *ATP1A1* subunit could protect from the cell death induced by Digoxin if this is part of its relevant senolytic target. In addition, the mouse is known to express a Na+/K+ATPase that is particularly resistant to the inhibitory action of CGs^34^. Thus, overexpression of *Atp1a1*, the mouse ortholog of the alpha 1 subunit of the Na+/K+ATPase, should show an even stronger effect protecting senescent cells from Digoxin-induced cell death. Lentiviral transduction of A549 cells with *ATP1A1*, *Atp1a1*, or GFP as a negative control, showed that indeed the overexpression of the alpha subunit of the Na+/K+ATPase pump protects cells from the senolytic effect of Digoxin (Fig. 3b). This protection was stronger when the mouse subunit was expressed (Fig. 3b), even when similar levels of overexpression were induced (Fig. 3c).

Interestingly however, the levels of expression of *ATP1A1* are very similar between proliferating and senescent cells, suggesting that differential expression of *ATP1A1* is not the cause behind the higher sensitivity of senescent cells to Digoxin (Supplementary Fig. 3a). To further confirm the involvement of *ATP1A1* as the relevant senolytic target of Digoxin, we knocked down this subunit by siRNA transfection. However, low levels of *ATP1A1* did not result in cell death (Supplementary Fig. 3b), suggesting that perhaps other alpha subunits of the Na+/K+ATPase pump might also be involved.

Inhibition of the Na+/K+ATPase pump by CGs have an obvious effect on the relative intracellular concentration of Na+ and K+ions that can lead to the alteration of the plasma membrane potential (Fig. 3a). Using a fluorescent probe (DiBAC4(3)), we measured plasma membrane potential in proliferative and senescent cells, treated or not with Digoxin. Senescent cells showed a slight but significant increase in the fluorescent signal of DiBAC4(3) compared with proliferating cells, an indication of a partially depolarized plasma membrane in senescent cells (Fig. 3d). This signal was further increased when senescent cells were treated with Digoxin, a clear indication of the depolarizing effect of Digoxin on senescent cells (Fig. 3d). To alleviate the depolarization of the plasma membrane caused by the altered ratio of Na+/K+, we decided to increase the concentration of K+ in the culture medium by adding KCl. Addition of KCl to the culture medium recovered the normal polarization of the plasma membrane as expected (Fig. 3d) while at the same time rescued the senolytic cell death induced by Digoxin (Fig. 3e). In turn, further increasing the concentration of Digoxin overcame the protective effect of KCl (Fig. 3e). These results suggest that depolarization of the plasma membrane by CGs could, at least in part, explain their senolytic effect.

The increased concentration of sodium as a result of the Na+/K+ATPase pump inhibition by CGs can result in the blockade of the Na+/Ca2+ Exchanger (Supplementary Fig. 3c) and the Na+/H+ Exchanger (Fig. 3a) due to the high intracellular concentration of Na+^39,40^. As a consequence, the levels of intracellular Ca2+ and H+ could increase and trigger cell death. We decided to measure intracellular Ca2+ levels using a fluorescent probe, Calcium-6. Senescent cells showed a higher concentration of intracellular Ca2+ compared to proliferating cells, and Digoxin treatment increased even further this high level of Ca2+ in senescent cells but not in proliferating cells (Supplementary Fig. 3d). To test whether the raise in intracellular Ca2+ could be behind the senolytic effect of Digoxin we treated proliferating and senescence cells with a Ca2+ chelator, BAPTA-AM. Senescent cells treated with Digoxin showed cell death independently of the presence or not of BAPTA-AM, suggesting that intracellular Ca2+sequestration does not have a protective effect against Digoxin-induced senolysis (Supplementary Fig. 3e). On the other hand, to measure the intracellular concentration of H+ we used a fluorescent probe, pHrodo Green AM, that turns from weakly to strongly fluorescent as the pH drops. Senescent cells showed a strong fluorescent signal compared to proliferating cells even in the absence of Digoxin, but CG treatment caused an even further increase in the fluorescence signal shortly (6 h) after treatment (Fig. 3f), indicating a decrease in pH before senescent cells undergo cell death. A prediction from this result is that pharmacological inhibition of the NHE, using for example Amiloride, should phenocopy the effect of Digoxin on senescent cells. We treated proliferative and senescent cells with increasing concentrations of Amiloride or left the cells untreated and measured cell viability. Senescent cells were significantly more affected and showed reduced viability, mimicking Digoxin senolytic action (Fig. 3g). Conversely, increasing NHE activity should have protective effect against the senolytic action of Digoxin. To test this possibility, we overexpressed NHE1 (encoded by the gene *SLC9A1*) in proliferating and senescent cells and treated the cell cultures with Digoxin. While proliferating and senescent cells showed similar levels of overexpression of *SLC9A1* (Fig. 3h), senescent cells treated with Digoxin were more protected by *SLC9A1* than the control (Fig. 3i).

Taken together, our data point to the alpha subunit of the Na+/K+ATPase pump as the relevant target in Digoxin-induced senolysis. Digoxin, by inhibiting this Na+/H+ Exchanger causes an imbalance in the cellular concentrations of sodium and potassium that results in the loss of membrane potential and acidification of the cell. This effect is particularly detrimental in senescent cells that already show depolarization of their plasma membranes and high concentration of H+ in resting conditions.

### Digoxin cooperates with senogenics in tumor regression

**Fig. 4:**
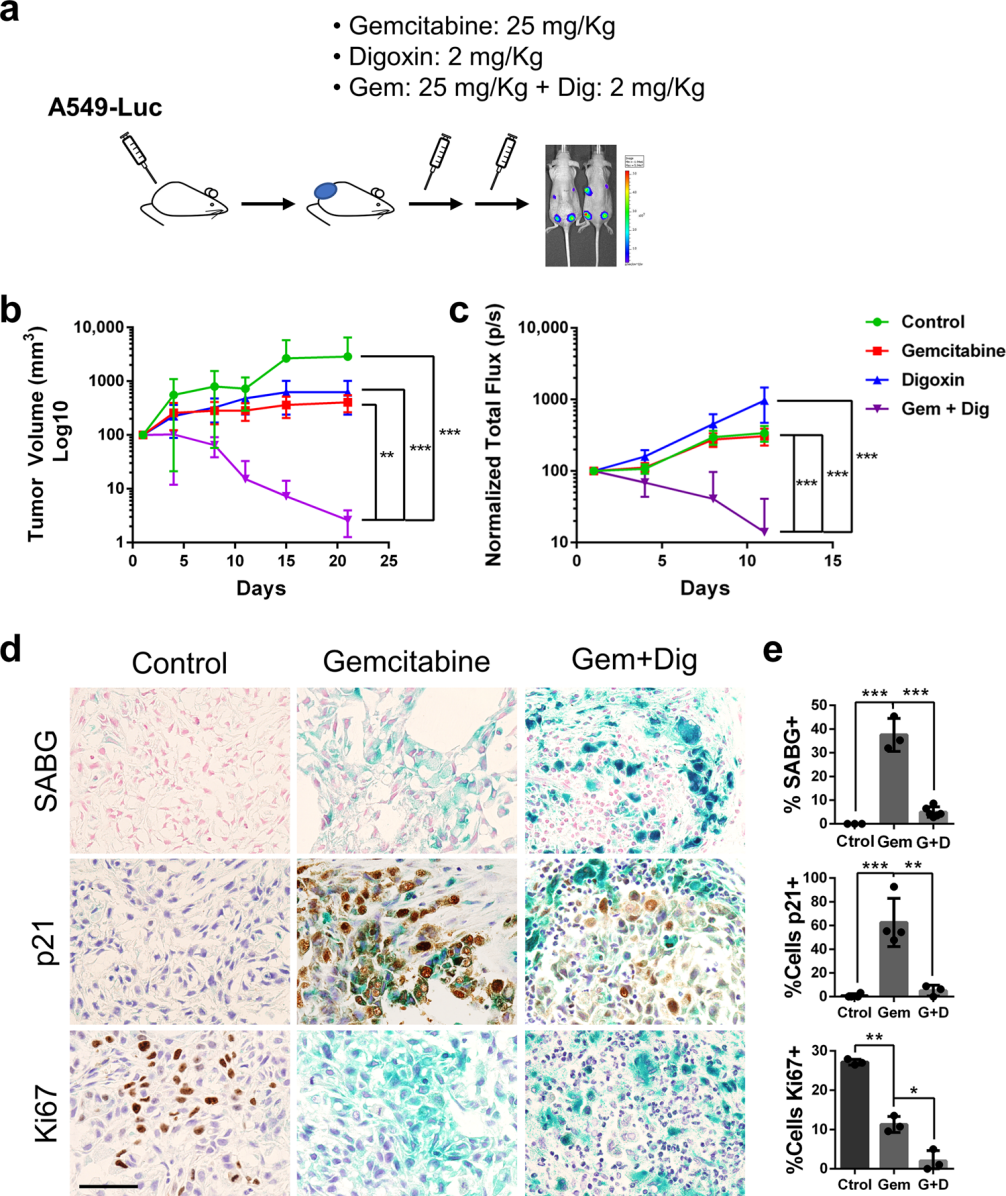
Senolytic activity of Digoxin on xenografts. **a** Diagram showing the experimental plan to test the effect of administrating Gemcitabine, Digoxin or the combination of both (Gem+Dig) in the growth of A549 cells expressing luciferase (A549-Luc) as subcutaneous tumors in nude mice. **b** Tumor volume (mm3) progression with time after intraperitoneal injection of Gemcitabine (*n* = 12), Digoxin (*n* = 8) or the combination of both (Gem+Dig) (*n* = 12), or vehicle (*n* = 4) as negative control as indicated. Statistical analysis was performed with Anova with Tuckey test: ***p < 0.001; **p < 0.01. **c** Tumor growth determined by measuring normalized total flux of luminescence as detected by IVIS with the same groups of animals. Statistical analysis was performed with Anova with Tuckey test: ***p < 0.001. **d** Representative images of SABG staining (upper panels) and immunohistochemical analysis of p21 (middle panels) and Ki67 (bottom panels) in tumors obtained after injection of A549 cells in nude mice and treated with Gemcitabine, Gemcitabine plus Digoxin (Gem+Dig), or control group (scale bar = 50 μm). **e** Quantifications of stainings shown in (**d**). *n* = 3 biologically independent samples. Statistical significance was assessed by the two-tailed Student's t-test: ***p < 0.001; **p < 0.01; *p *<* 0.05. All data correspond to the average ± s.d. Source data for these experiments are provided as a Source Data file

One of the settings in which administration of a senolytic drug could have a clear beneficial effect is chemotherapy of cancer. Irreversibly arresting tumor cells with a senescence-inducing drug combined with inducing cell death with a senolytic compound could potentially prove superior than the classical antitumor chemotherapy. Our screenings for senolytic compounds were all based on senescent tumor cells, both lung adenocarcinoma A549 and melanoma SK-MEL-103. To address whether CGs could have antitumor activity by providing senolysis to a senescence-inducing drug we injected subcutaneously A549 cells stably expressing luciferase into immunodeficient nude mice, we let the cells form tumors and, once they were detectable, we started a treatment regime. For this, we administered Gemcitabine (25 mg/kg, IP) or Digoxin (2 mg/kg, IP) alone, or the combination of Gemcitabine plus Digoxin, and followed tumor progression by measuring tumor volume and luciferase expression, twice weekly (Fig. 4a). Both, Gemcitabine and Digoxin alone had a slight antitumor effect as shown by the reduced growth of the tumor cells compared to the control animals (Fig. 4b, c). Interestingly, the combination of Gemcitabine and Digoxin showed a robust antitumor effect with a dramatic drop on tumor volume and luciferase signal along the treatment period, with most of the tumors actually disappearing after three weeks of treatment (Supplementary Fig. 4a). Immunohistochemical analysis of the treated tumors showed how Gemcitabine induces a robust senescent response in the tumor as observed from the positive senescence-associated beta-galactosidase (SABG) staining, the loss of proliferation marker Ki67, and the expression of cell cycle inhibitor p21 (Fig. 4d, e), all of them markers of cell senescence^41^. Combination of Gemcitabine with Digoxin resulted in extremely reduced tumors that were mainly composed of macrophages and giant multinucleated immune cells surrounding a few SABG and p21 positive, non-proliferating CK AE1/AE3 positive tumor cells (Fig. 4d and Supplementary Fig. 4b). We also stained these samples for active Caspase-3 and only observed very few positive tumor cells undergoing apoptosis in the combination Gemcitabine plus Digoxin (Supplementary Fig. 4c, d).

**Fig. 5:**
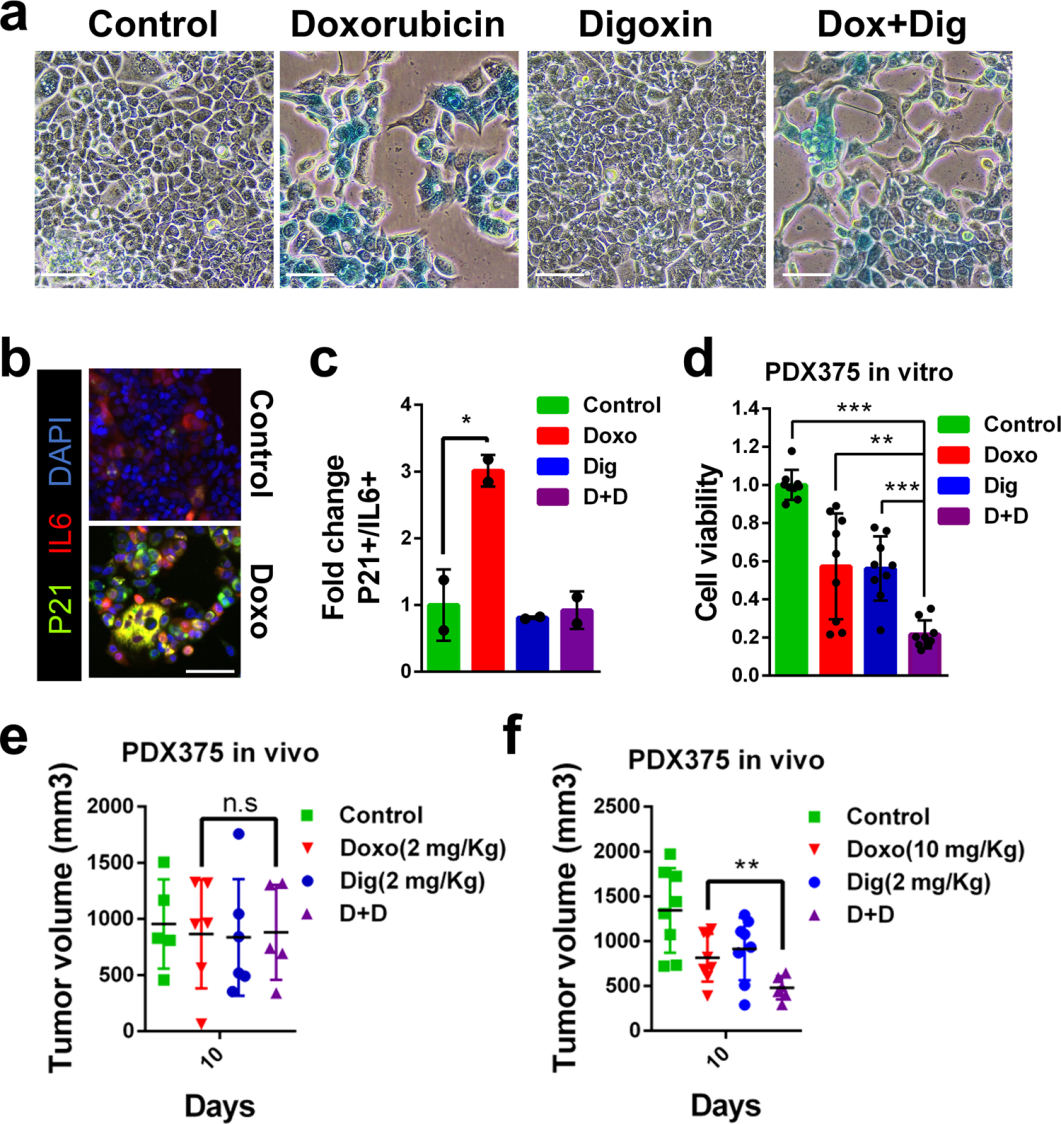
Senolytic activity of Digoxin on human PDX. **a** In vitro SABG staining of PDX375 in control, Doxorubicin, Digoxin and Doxorubicin plus Digoxin (Dox+Dig) treated cells (scale bar = 100 μm). **b** Representative immunofluorescent images of PDX375 cells transduced with lentiviral reporter constructs for the expression of p21 (in green) and IL6 (in red) in control condition or after Doxorubicin treatment. DAPI (in blue) was used to stained nuclei (scale bar = 100 μm). **c** Quantification (fold change) of double positive p21/IL6 cells in control, or Doxorubicin (Doxo), Digoxin (Dig), or Doxorubicin plus Digoxin (D+D) treatment (*n* = 3 biologically independent samples). **d** In vitro cell viability (relative to control) of PDX375 cells treated with Doxorubicin (Doxo), Digoxin (Dig), or the combination (D+D) (*n* = 3 biologically independent samples). **e** In vivo growth (tumor volume) of PDX375 subcutaneously injected into nude mice and treated with Doxorubicin (Doxo: 2 mg/kg; *n* = 6), Digoxin (Dig: 2 mg/kg; *n* = 6), the combination (D+D; *n* = 5), or vehicle (Control; *n* = 5). **f** Same in vivo growth analysis in mice treated with Doxorubicin (Doxo: 10 mg/kg; *n* = 8), Digoxin (Dig: 2 mg/kg; *n* = 8), the combination (D+D; *n* = 6), or vehicle (Control; *n* = 8). Statistical significance was assessed by the two-tailed Student's t-test: ***p < 0.001; **p < 0.01; *p *<* 0.05. All data correspond to the average ± s.d. Source data for these experiments are provided as a Source Data file

To further prove the contribution of the senolytic activity provided by Digoxin to the control of tumor growth we decided to use a patient derived xenograft obtained from a breast tumor patient (PDX375). This PDX was first characterized in vitro to test its response to the chemotherapy agent Doxorubicin. Staining with SABG showed a clear induction of cell senescence and a decreased number of cells when Doxorubicin was used but not when cells were treated with Digoxin (Fig. 5a). In addition, we transduced the tumor cells with reporter constructs for the expression of *CDKN1A* (coding for p21) and *IL6*, both considered markers of senescence^41^. Treatment with Doxorubicin caused a clear induction of the reporters with some cells staining positive for both markers, indicative of senescence (Fig. 5b, c). When we treated the cells with Doxorubicin or Digoxin alone the viability was slightly reduced, while the combination of Doxorubicin and Digoxin resulted in a pronounced decrease in survival (Fig. 5d). Next, we injected PDX375 in nude mice and treated the animals with either compound alone or the combination, and we determined tumor growth. Doxorubicin treatment was performed at two different concentrations. When we treated mice with a lower concentration (2 mg/kg), unable to induce senescence, Digoxin did not show any collaborative effect (Fig. 5e). In contrast, mice treated at a senescence-inducing concentration of Doxorubicin (10 mg/kg) showed a significant reduction in tumor volume when treated together with Digoxin (Fig. 5f). In these models we treated human cancer cells implanted into immunodeficient mice. Unfortunately, the known insensitivity of rodents to CGs^34^ precludes the possibility of using immunocompetent mouse models of cancer development.

In summary, we have shown that Digoxin has antitumor properties when combined with different senogenic chemotherapeutic agents using two in vivo mouse models.

### Digoxin is senolytic in lung fibrosis

**Fig. 6:**
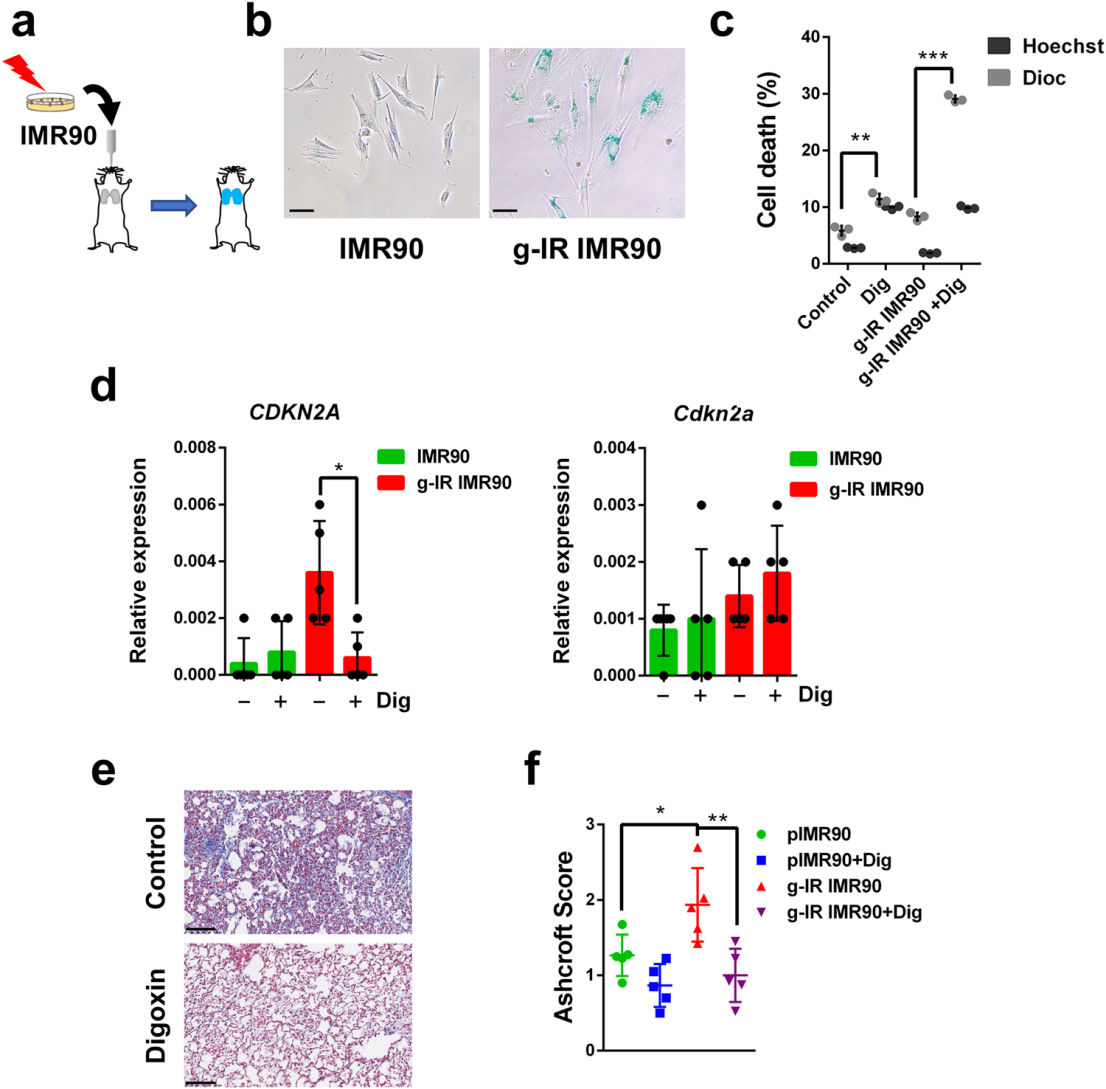
Senolytic activity of Digoxin in lung fibrosis. **a** Schematic diagram of the experimental system to induce lung fibrosis in mice by intratracheal instillation of proliferative or senescent gamma-irradiated IMR90 cells. **b** In vitro SABG staining of control or gamma-irradiated (g-IR) IMR90 cells (scale bar = 100 μm). **c** In vitro analysis of cell death in control or (g-IR) IMR90 cells treated or not with Digoxin (Dig). Black bars represent the % of DiOC6(3) low and grey bars represent Hoechst 33342 positive cells. *n* = 3 independent experiments. **d** Relative expression of the mRNA coding for *CDKN2A* (left panel) or *Cdkn2a* (right panel) in lung cell extracts from mice injected with control proliferative (green) or gamma-irradiated (g-IR, red) IMR90 cells, and treated or not with Digoxin, as indicated. *n* = 5 independent experiments. **e** Representative images of lung sections stained with Masson Trichrome from mice injected with gamma-irradiated IMR90 cells and treated (bottom panel) or not (upper panel) with Digoxin (scale bar = 100 μm). **f** Ashcroft score of Masson Trichrome staining in sections from mice injected with control proliferative or gamma-irradiated (g-IR) IMR90 cells, treated or not with Digoxin (+Dig). *n* = 5 independent experiments. Statistical significance was assessed by the two-tailed Student's t-test: ***p < 0.001; **p < 0.01; *p < 0.05. Data correspond to the average ± s.d. Source data for these experiments are provided as a Source Data file

Next, we wanted to test the senolytic effect of Digoxin using another in vivo model of a non-tumor related disease. For this, we established a mouse model of lung fibrosis induced by intratracheal administration of senescent human cells. Normal proliferating or gamma-irradiated (g-IR) senescent human fibroblasts IMR90 were delivered into the lungs of immunodeficient mice (Fig. 6a, b). Cell death analysis of in vitro treatment with Digoxin of proliferating and g-IR IMR90 confirmed once again the senolytic effect of the CG (Fig. 6c). Three weeks after intratracheal instillation, these animals were subjected to Digoxin or vehicle treatment for ten days and their lungs were removed and analyzed. First, we measured the expression levels of *CDKN2A* (coding for p16INK4a) a typical marker of primary fibroblast senescence^42^. Lungs from animals injected with g-IR IMR90 showed high levels of this human gene but not of the mouse ortholog, *Cdkn2a*, indicative of the presence of senescent human cells in the mouse lungs (Fig. 6d). Similarly, human *CDKN1A* (coding for p21) was also detected in the lungs of mice receiving senescent human cells (Supplementary Fig. 5a). Treatment with Digoxin caused a significant reduction in *CDKN2A* expression levels and of *CDKN1A* (although not reaching statistical significance for this gene), suggesting that Digoxin caused the clearance of the human senescent cells (Fig. 6d and Supplementary Fig. 5a). Finally, we stained the lungs with Masson Trichrome, a well-established marker of fibrosis. Lungs from animals injected with senescent g-IR IMR90 and treated with vehicle stained positive, indicative of fibrosis, while those treated with Digoxin had a reduced score of fibrosis (Fig. 6e, f). In addition, we determined hydroxyproline content in lungs as a measure of fibrosis and found that animals receiving senescent g-IR IMR90 cells had a significantly higher level of this marker compared to the ones injected with proliferating cells (Supplementary Fig. 5b). In line with our results using Masson Trichrome staining, mice treated with Digoxin showed a tendency to have reduced levels of hydroxyproline (Supplementary Fig. 5b).

These results show the potential senolytic effect exerted by Digoxin in vivo using a model of a disease, other than cancer, caused by the accumulation of senescent cells.

## Discussion

In recent years, the field of cell senescence has experienced a significant expansion in part due to robust in vivo proof of the causative involvement of senescent cells in aging and in particular age-related diseases^10,11^. One of the elements providing a decisive support for this recognition is the identification of senolytics, small molecules with selective cytotoxic activity on senescent cells^43^. Senolytics represent powerful tools to unequivocally demonstrate the involvement of senescence cells in diseases characterized by their accumulation^44^. At the same time, they hold the promise of novel and effective treatments against age-related diseases^27,45,46^.

The use of cellular senescence as a therapeutic tool was pioneered by efforts trying to use it as an antitumor strategy given the powerful tumor suppressive function of this response^13^. However, the prospect of letting senescent cancer cells hanging around in the organism represents a potential threat of developing a secondary tumor with increased aggressiveness. The identification and description of the complex secretory phenotype characteristic of senescent cells known as SASP, with potential tumor promoting activities, raised even more suspicion about the possible harmful consequences of inducing senescence in tumor cells^18^. In our view, combining senogenic and senolytic compounds could have a synergistic antitumor effect that could prove very effective in cancer treatment^47^. With these ideas in mind we decided to search for new senolytic compounds that could offer us the possibility of identifying vulnerabilities in senescent cells and could provide us with novel therapeutic tools against age-related diseases and in particular, cancer.

Cell-based phenotypic screening of chemical libraries represents a powerful tool to identify new potential drugs^48^. We designed a robust screening platform for senolytic compounds and tested it using a chemical library of approved drugs. As a result of this screening we identified a compound, Proscillaridin A, a drug belonging to the Cardiac Glycoside family. Interestingly, two additional screenings using a library of compounds derived from plant extracts and a collection of venoms, also identified hits belonging to this same CGs family of compounds. Digoxin, one of these CGs, is a commonly used drug for atrial fibrillation and heart failure and in our hands showed a powerful senolytic activity. Even though the therapeutic window for Digoxin is quite narrow, this is a drug that has been successfully used for decades. We can benefit from our knowledge on how to administer the drug and how to follow and assess the patients, and we have even developed effective blocking monoclonal antibodies to revert possible drug overdoses^49^. Our experiments show that at least for some particular cell types and senescence inducers, the doses needed to achieve the senolytic effect fall within the therapeutic range. This might not be the case for all the cell types and an individual assessment will have to be done to develop potential particular therapies.

Even though our screenings were based on the use of human cancer cells, A549 and SK-MEL-103, induced to senescence either by Bleomycin or Palbociclib, we showed that the use of other chemotherapeutic drugs to induce senescence also allows senolysis by Digoxin. In addition, primary BJ fibroblasts induced to senescence by Bleomycin, expression of an oncogene, or damaged by oxidative stress were also more sensitive to the cytotoxic effect of Digoxin than their proliferative counterparts. We have also expanded these observations to other tumor and primary cells. In addition, similar results have been obtained by another group performing independently cell-based screenings and using other unrelated chemical libraries, further reinforcing the reproducibility of our findings^50^.

CGs are known to exert their action primarily by binding to the alpha subunit of the Na+/K+ATPase pump and inhibiting the intake of K+ coupled with the release of Na+out of the cell^34^. This inhibition initiates a cascade of events that results in distorted electrochemical gradients involving several ions within the cell. We confirmed the depolarization caused by Digoxin and found that senescent cells show a slight depolarized plasma membrane that puts these cells closer to a level of depolarization that can trigger cell death when treated with Digoxin. Incubation of senescent cells with KCl was sufficient to protect these cells from senolysis induced by Digoxin and overexpression of the alpha 1 subunit of the Na+/K+ATPase also partially rescued cell death, proving the relevance of the known CG target on this effect. The increased levels of Na+within the cell can result in the blockade of the Na+/H+Exchanger, causing an increase in the H+concentration. Indeed, the high concentration of H+seems to be involved in Digoxin senolytic effect. Amiloride-mediated inhibition of NHE phenocopied the effect of Digoxin, while overexpression of NHE1 protected senescent cells from senolysis. Interestingly, the levels of H+in senescent cells were already higher than in proliferating cells and Digoxin further increased this concentration.

As already mentioned above, we support the possibility of developing a two-punch strategy against cancer based on cellular senescence by combining senogenic and senolytic drugs. Since we based our screening on the use of human cancer cells induced to senescence by chemotherapeutic drugs, we decided to test the effectiveness at controlling tumor growth of our candidate senolytic, Digoxin. For this, we used xenografted A549 cells or a breast cancer PDX treated with senogenic compounds Gemcitabine or Doxorubicin, respectively. Mice were treated with the senogenics alone or in combination with Digoxin. Compared to the weak antitumor effect obtained with each compound alone, the combination of Gemcitabine or Doxorubicin plus Digoxin proved to be synergistic and efficient at reducing the growth of the cancer cells in vivo and even inducing complete regression of tumors. To extend the range of potential applications of CGs as senolytics, we tested a mouse model of lung fibrosis triggered by administration of senescent human fibroblasts directly into the lung. In this system, Digoxin preferentially induced cell death of senescent cells and reduced the extend of the fibrosis.

In summary, we have identified Cardiac Glycosides as senolytic compounds with robust activity both in vitro and in vivo, and effective against tumor and primary cells. Depolarization and high H+concentrations in senescence cells represent a vulnerability that can be exploited for therapy. Cardiac Glycosides, by inducing a disbalanced gradient in sodium and potassium, provide a crucial push into the abysm of cell death for senescent cells. Our findings contribute to age-related disease drug discovery by identifying senolytic compounds and demonstrating potential for therapeutic application.

## Methods

### Cells and reagents

All cell lines were obtained from the ATCC. Human breast tumors used to establish Patient-Derived Xenografts (PDXs) in this study were from biopsies or surgical resections at Vall d’Hebron University Hospital, Barcelona, and were obtained following institutional guidelines. The IRBs at Vall d’Hebron Hospital provided approval in accordance with the Declaration of Helsinki. Written informed consent was obtained from all patients who provided tissue.

Cells were cultured in DMEM (Sigma) supplemented with 10% FBS (Sigma) and were routinely tested for mycoplasma contamination. Navitoclax (ABT-263, Abbvie) was used as standard senolytic reagent. For chemotherapy-induced senescence we used Bleomycin (Mylan pharmaceuticals) at 20 μM for 5 days. Alternatively, we also used 1 µM Gemcitabine (Hospira UK), 1 µM Doxorubicine (TEDEC-MEIJI), 1 µM Etoposide (TEVE Genericos Española), and 5 µM Palbociclib (Goodchem). For Cardiac Glycosides we used Ouabain (Sigma), Digoxin (Kern Pharma) and Proscillaridin A (Sigma). Amiloride was from Sigma. The chemical libraries used for screening were: Prestwick Chemical Library (Prestwick Chemical), GPNCL library of natural compounds (Greenpharma Natural Chemical Library), SCREEN-WELL^®^ Natural Product library (ENZO). For membrane depolarization we used DiBAC4(3) fluorescent probe (Thermofisher), and pHrodo Green to measure the relative H+concentration.

### IC50 calculation

IC50s were calculated using cell cultures induced to senescence by different stimuli (chemotherapy, RAS expression or exposure to H_2_O_2_) and subsequent treatment with the senolytic compounds starting at a high concentration and using serial dilutions of the compounds. Cells were counted using a High-Content Imaging System (Operetta, Perkin Elmer).

### Apoptosis analysis

After treatments, cells were stained for Annexin-V-FITC or cleaved Caspase-3-PE using commercial kits (Immunostep and BD Biosciences, respectively) following the protocols from the providers. Positive cells were determined using FACScalibur (BD Biosciences). To block apoptosis induction, we used pan-caspase inhibitor Z-VAD-FMK (Santa Cruz Biotechnology) 10 mM for 24 h. In the case of proliferating and g-IR IMR90 cells we used DiOC6(3) to measure the loss of mitochondrial potential. Cell death was monitored by summing the % of DioC6(3) low and Hoechst 33342 positive cells.

### In vivo senolytic activity

Animal procedures testing senolytic activity on xenografted A549 cells were approved by University of Santiago de Compostela Bioethics Committee in compliance with Principles of Laboratory Animal Care of national laws (license number 15010/17/001). Female 8 weeks old immunodeficient nude NMRInu/nu mice (Janvier) were subcutaneously injected under isofluorane anesthesia with 1 × 10^6^ A549-luc cells. When tumors were detectable, mice were injected intraperitoneally with Gemcitabine (25 mg/Kg; Hospira UK), Digoxin (2 mg/kg; Kern Pharma), or vehicle (PBS), twice weekly. Tumor growth was followed by measuring their volume using a caliper and the formula V = (W(2) × L)/2, and by luminescence imaging using IVIS Spectrum in vivo imaging system (Perkin Elmer). Care and use of animals to test senolysis in human PDX375 at Vall d’Hebron Campus strictly complied with European, Spanish and Catalan Regulations for Protection of Vertebrate Animals for Experimental and Scientific Purposes. Studies were performed in an authorized establishment (Lab Animal Service Campus Vall d’Hebron (license number B9900062). Experimental procedure was essentially identical, with Doxorubicin used as senogenic compound (10 mg/kg). For the lung fibrosis experiments, protocols were approved by the Ethical Committee for Animal Experimentation (CEEA) of the Scientific Park of Barcelona (PCB license number CEEA-19-029) and the Government of Catalunya and complied with their ethical regulations. Intratracheal administration of 1 × 10^6^ IMR90 cells was followed by Digoxin (2 mg/kg) or vehicle treatment. Lungs were removed and analyzed by staining for Masson Trichrome or used to extract RNA for QPCR.

### Gene expression

Retroviral or lentiviral transduction was used to overexpress the following genes: GFP as control (from FUGW, a gift from David Baltimore; Addgene plasmid #14883)^51^, *Hras*V12 (from pBabe-puro-*Hras*V12, a generous gift of Matthias Drosten, CNIO, Madrid, Spain); *Atp1a1*, *ATP1A1* and *SLC9A1* (a generous gift from Sean Morrison, UTSW, Dallas, USA)^52^. To obtain stable A549 cells expressing GFP or RFP for screening, we used pLJM1-EGFP and pLJM3-RFP (a kind gift from Alejo Efeyan, CNIO, Madrid, Spain). We used the green reporter vector of System Bioscience (SR010PA-hp21) to follow the expression of the human *CDKN1A*. To construct an *IL6* reporter we cloned 1.1 kb of the human *IL6* promoter into the empty pRZ reporter vector (SR10046), amplified by PCR and cloned with ClaI and BamHI. For transduction, we co-transfected HEK293T cells (5 × 10^6^ cells per 100-mm-diameter dish) with the plasmid of interest and the corresponding packaging vector, the ecotropic pCL-Eco or the third-generation system: pLP1, pPL2, pLP-VSVG, respectively, using PEI reagent. Viral supernatants were serially collected 3 times every 12 hours, starting 36 h after transfection. The supernatants were filtered through a 0.45 μm filter and polybrene (Sigma) was added to a final concentration of 8 μg/mL. The target cells were plated (1.4 × 10^6^ cells per 100-mm-diameter dish) the day before to the first round of transduction.

### Q-RT-PCR

To measure RNA expression, total RNA was extracted using the NucleoSpin RNA kit (Macherey-Nagel) according to the manufacturer’s instructions. After quantification of RNA in a nanodrop, the RNA was retrotranscribed into cDNA with High-Capacity cDNA-Reverse Transcription Kit (Applied Biosystems). Quantitative Real Time-PCR was performed using SYBR Green Power PCR Master Mix (Applied Biosystems) in an AriaMx Real-Time PCR system (Agilent Technologies). Relative RNA expression was normalized using the housekeeping gene *GAPDH*. Primer sequences are:

*ATP1A1-*F: 5′-ACAGACTTGAGCCGGGGATTA-3′

*ATP1A1-*R: 5′-TCCATTCAGGAGTAGTGGGAG-3′

*Atp1a1*-F: 5′-ACATTCCGGAAATCACCCCC-3′

*Atp1a1*-R: 5′-CCCGTACACGGTGTTTCTCA-3′

*GAPDH*-F: 5′-TCCATGACAACTTTGGCATCGTGG-3′

*GAPDH*-R: 5′-GTTGCTGTTGAAGTCACAGGAGAC-3′

*SLC9A1*-F: 5′-ACCACGAGAACGCTCGATTG-3′

*SLC9A1*-R: 5′-ACGTGTGTGTAGTCGATGCC-3′

*Cdkn2a*-F: 5′-TACCCCGATTCAGGTGAT-3′

*Cdkn2a*-R: 5′-TTGAGCAGAAGAGCTGCTACGT-3′

*CDKN2A*-F: 5′-GATCCAGGTGGGTAGAAGGTC-3′

*CDKN2A*-R: 5′-CCCCTGCAAACTTCGTCCT-3′.

### Senescence-associated β-Galactosidase staining

Senescence-associated β-Galactosidase (SABG) staining was performed following well-described methods^53^. Basically, cells or tissues were fixed at room temperature in 2% formaldehyde/0.2% glutaraldehyde, washed, and incubated overnight at 37 °C with fresh SABG staining solution: 1 mg of 5-bromo-4-chloro-3-indolyl beta-D-galactoside (X-Gal) per mL (Fisher Scientific), 40 mM citric acid/sodium phosphate pH 6.0, 5 mM K_3_Fe[CN]_6_, 5 mM K_4_Fe[CN]_6_, 150 mM NaCl, and 2 mM MgCl_2_. Cells were washed and visualized and photographed under the microscope. Tissues were embedded in paraffin, sectioned and counterstained with Nuclear Fast Red or further used for immunohistochemistry.

### Immunohistochemical analysis

Whole mount SABG stained tissues were sectioned and slides were incubated overnight at 37 °C before being deparaffinized in xylene and rehydrated in a descending series of ethanol solutions. Antigen retrieval was carried out in a PTLink instrument (Dako) and immunoreactive cells were visualized using 3,3-diaminobenzidine tetrahydrochloride plus (DAB+) as a chromogen. Sections were incubated with antibodies against proliferative marker Ki67 (prediluted SP6, Master Diagnostica 0003110QD) and senescence mediator p21Cip1 (HUGO-291 CNIO, 1:10 dilution).

### Statistical analyses

For in vivo studies, mice were randomly assigned to treatment groups; sample size was not pre-determined. A two-tailed unpaired Student's t-test was applied for comparison between groups; all analyzed samples were included for statistical analysis. For some analyses we used Anova with Tuckey test as indicated. All the experiments were repeated at least three times, except the in vivo experiment with mice, and a single representative experiment is shown. All measurements were performed at least in triplicate. Statistical analyses and graphical representations were performed with GraphPad Prism 7.0.

More technical details can be found in the Supplementary Methods section accompanying this paper.

### Reporting summary

Further information on research design is available in the Nature Research Reporting Summary linked to this article.

## Supplementary information

Supplementary information

Reporting Summary

## Data Availability

The data that support the findings of this study are available from the corresponding authors upon reasonable request. The source data underlying Figs. 1c–f, 2a–c, 3b–i, 4b, c, e, 5c–f, 6c–f and Supplementary Figs. 1a, b, c, e, f, g, 2, 3a, b, d, e, 4d, and 5a, b are provided as a Source Data file.

## Source data

Source Data
