# Author Correction: Identification and characterization of Cardiac Glycosides as senolytic compounds

**DOI:** 10.1038/s41467-020-18714-z

**Published:** 2020-09-16

**Authors:** Francisco Triana-Martínez, Pilar Picallos-Rabina, Sabela Da Silva-Álvarez, Federico Pietrocola, Susana Llanos, Verónica Rodilla, Enrica Soprano, Pablo Pedrosa, Alba Ferreirós, Marta Barradas, Fernanda Hernández-González, Marta Lalinde, Neus Prats, Cristina Bernadó, Patricia González, María Gómez, Maria P. Ikonomopoulou, Pablo J. Fernández-Marcos, Tomás García-Caballero, Pablo del Pino, Joaquín Arribas, Anxo Vidal, Miguel González-Barcia, Manuel Serrano, María I. Loza, Eduardo Domínguez, Manuel Collado

**Affiliations:** 1 Laboratory of Stem Cells in Cancer and Aging, Health Research Institute of Santiago de Compostela (IDIS), Xerencia de Xestión Integrada de Santiago (XXIS/SERGAS), E15706 Santiago de Compostela, Spain; 2 BioFarma, Center for Research in Molecular Medicine and Chronic Diseases (CIMUS), Universidade de Santiago de Compostela, Health Research Institute of Santiago de Compostela (IDIS), Santiago de Compostela, Spain; 3 Institute for Research in Biomedicine (IRB Barcelona), The Barcelona Institute of Science and Technology (BIST), 08028 Barcelona, Spain; 4 DNA Replication Group, Spanish National Cancer Research Centre (CNIO), 28029 Madrid, Spain; 5 Preclinical Research Program, Vall d´Hebron Institute of Oncology (VHIO) and CIBERONC, Barcelona, Spain; 6 Centro Singular de Investigación en Química Biolóxica e Materiais Moleculares (CiQUS) and Departamento de Física de Partículas, Universidade de Santiago de Compostela, 15782 Santiago de Compostela, Spain; 7 Metabolic Syndrome Group, Madrid Institute for Advanced Studies (IMDEA) in Food, CEI UAM+CSIC, Madrid, E28049 Spain; 8 Department of Pulmonology, ICR, Hospital Clinic, Instituto de Investigaciones Biomedicas August Pi i Sunyer (IDIBAPS), Barcelona, 08036 Spain; 9 Histopathology Unit, Spanish National Cancer Research Centre (CNIO), 28029 Madrid, Spain; 10 Translational Venomics Group, Madrid Institute for Advanced Studies (IMDEA) in Food, CEI UAM+CSIC, Madrid, E28049 Spain; 11 Departamento de Ciencias Morfológicas, Facultad de Medicina. USC. Xerencia de Xestión Integrada de Santiago (XXIS/SERGAS), E15706 Santiago de Compostela, Spain; 12 Catalan Institution for Research and Advanced Studies (ICREA), 08010 Barcelona, Spain; 13 CiCLOn, Centro Singular de Investigación en Medicina Molecular y Enfermedades Crónicas (CIMUS), Universidade de Santiago de Compostela, Instituto de Investigación Sanitaria de Santiago de Compostela (IDIS), E15782 Santiago de Compostela, Spain; 14 Servicio de Farmacia, Xerencia de Xestión Integrada de Santiago (XXIS/SERGAS), E15706 Santiago de Compostela, Spain

**Keywords:** Senescence, Drug development

Correction to: *Nature Communications* 10.1038/s41467-019-12888-x, published online 21 October 2019.

The original version of this Article mistakenly included the following sentence in the Acknowledgement section:

P.P.-R. receives support from a program by the Deputacion de Coruña (BINV-CS/2019). This has now been corrected in both the PDF and HTML versions of the Article.

