# Supplementary material for: Identification and characterization of Cardiac Glycosides as senolytic compounds
Source: Nat Commun. 2019 Oct 21;10:4731. doi: 10.1038/s41467-019-12888-x (PMC6803708; doi:10.1038/s41467-019-12888-x)
Supplement: Supplementary file 1 — Supplementary information [file 41467_2019_12888_MOESM1_ESM.pdf]

SUPPLEMENTARY INFORMATION

Identification and characterization of Cardiac Glycosides as senolytic compounds

Triana-Martínez **et al.**

SUPPLEMENTARY METHODS

Venom library

To screen for senolytic compounds present in venoms we used a particular collection of 200 venoms and venom-derived peptides and compounds from snakes, spiders, toads, bees, centipedes, ants, octopus and lizards.

Calcium measurement

For intracellular calcium determination we used the FLIPR Calcium-6 Assay Kit (Molecular Devices) following manufacture´s instructions. BAPTA-AM was used as chelator.

Immunohistochemistry

After deparaffination and epitope retrieval (in EnVision FLEX target retrieval solution at high pH, for 20 min at 97ºC), immunohistochemistry was automatically performed using an AutostainerLink 48 immunostainer (Agilent, Carpinteria, CA). Briefly, the slides were incubated at room temperature in: 1) Prediluted flex monoclonal antibody to CKAE1AE3 (Dako-Agilent, GA05361-2) for 30 min, or rabbit monoclonal antibody to Cleaved caspase 3 (clone 5A1E) (Cell Signaling, Danvers, MA, #9664; 1:300 dilution) for 20 min; 2) EnVision FLEX/HRP (dextran polymer conjugated with horseradish peroxidase and affinity-isolated goat anti-mouse immunoglobulins) for 20 min; 3) substrate working solution (mix) (3,3’ diaminobenzidine tetrahydrochloride chromogen solution) for 10 min; and 4) EnVision FLEX hematoxylin for 9 min.

Cell death analysis

To check the involvement of Ferroptosis and Necroptosis on Digoxin-induced cell death of senescent cells, we treated Bleomycin-induced senescent A549 cells with Digoxin (0.1 μM) alone, or in combination with Ferrostatin-1 (10 μM; Sigma) or Necrostatin-1

(20 μM; Sigma) for 24h, and cell viability was determined by cell counting using High-Content Imaging System (Operetta; Perkin Elmer).

Oligonucleotides for QPCR

Cdkn1a-F: 5'-GCAGATCCACAGCGATAT-3'

Cdkn1a-R: 5'-GGAACAGGTCGGACATCA-3'

CDKN1A-F: 5'-TGTCCGTCAGAACCCATG-3'

CDKN1A-R: 5'-TGCCTCCTCCCAACTCATC-3'

Hydroxyproline assay

After euthanasia, lungs were snap-frozen in liquid nitrogen. Lungs were then resuspended in PBS and homogenates were prepared by means of a tissue homogenizer (Fats Prep-24 5G, MP Biomedical). 10 mg of lung homogenates were used per assay. Total collagen levels were quantified using a Hydroxyproline Assay Kit (#6017, Chondrex) as indicated by the manufacturer.

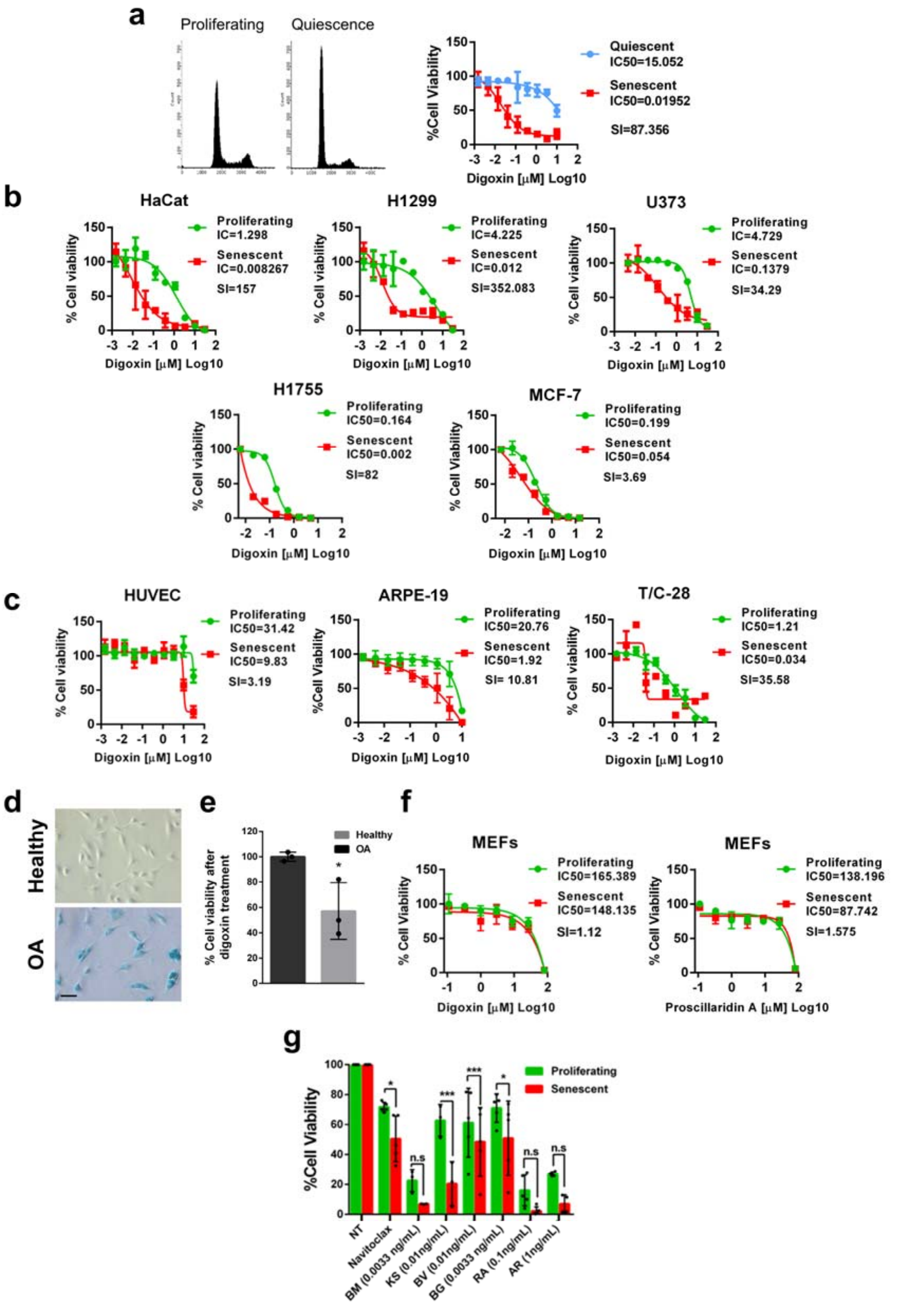

Supplementary Figure 1. (**a**) Cell cycle profile curves of proliferating and confluent, non-proliferating A549 cells, showing the accumulation of cells at G0/G1 phase (left

panel), and IC50 curves for Digoxin on quiescent and senescent cells (right panel). (**b**) IC50 curves obtained with increasing concentrations of Digoxin in (from left to right and top to bottom): HaCat, H1299, U373-MG, H1755, and MCF-7. Actual IC50 values and Senolytic Indexes (SI) are also shown for each condition. (**c**) IC50 curves obtained with increasing concentrations of Digoxin in primary HUVEC, ARPE-19 and T/C-28 cells. Actual IC50 values and Senolytic Indexes (SI) are also shown for each condition. (**d**) Representative images of healthy and osteoarthritic human chondrocytes stained for SABG (scale bar = 100 μm). (**e**) Relative cell viability (%) of healthy (grey bar) and osteoarthritic (black bar) human chondrocytes treated or not with Digoxin. n=3 biologically independent samples. Data correspond to the average ± s.d. Statistical significance was assessed by the two-tailed Student´s t-test: * $p < 0.05$. (**f**) IC50 curves obtained with increasing concentrations of Digoxin (left panel) or Proscillaridin A (right panel) in mouse embryo fibroblasts (MEFs). Actual IC50 values and Senolytic Indexes (SI) are also shown for each condition. (**g**) Relative cell viability (%) of melanoma SK-MEL-103 cells induced to senescence by Palbociclib (red) or actively proliferating (green) and treated with different extracts from toads (BM: *Bombina marinus*; KS: *Kassina senegalensis*; BV: *Bombina variegata*; BG: *Bombina garmani*; RA: *Rhinella arenarum*; AR: *Amietophrynus regularis*). n=3 independent experiments. Data correspond to the average ± s.d. Statistical significance was assessed by the two-tailed Student´s t-test: *** $p < 0.001$; * $p < 0.05$; *n.s.* > 0.05. Source data for these experiments are provided as a Source Data file.

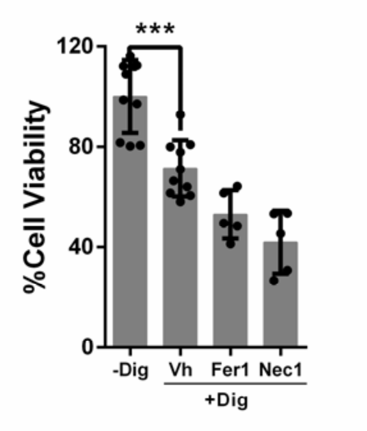

Supplementary Figure 2. Senescent A549 cell survival after Digoxin treatment alone (+Dig) or in combination with Ferrostatin-1 (Fer1) and Necrostatin-1 (Nec1), and control condition without Digoxin (-Dig). n=5 biologically independent samples. Data correspond to the average ± s.d. Statistical significance was assessed by the two-tailed Student´s t-test: *** $p < 0.001$. Source data for these experiments are provided as a Source Data file.

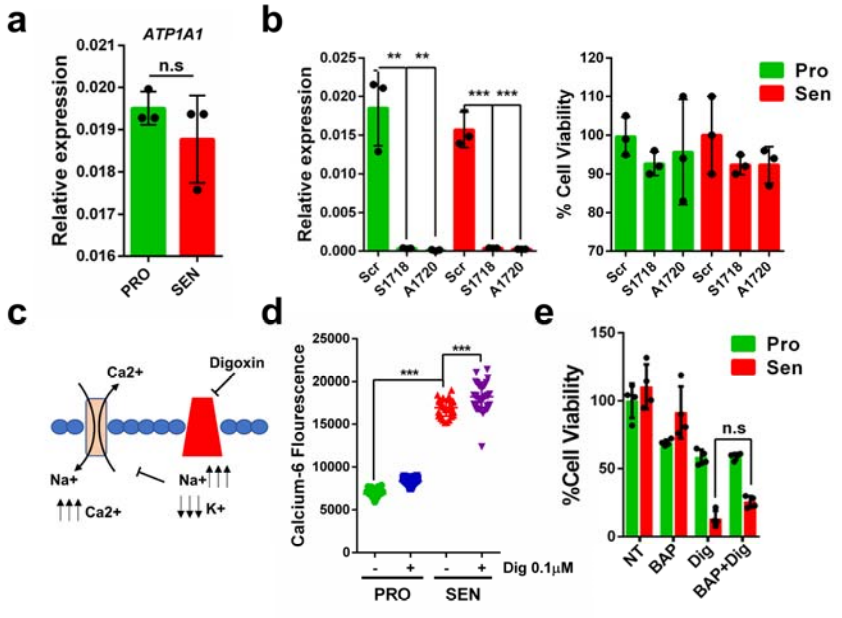

Supplementary Figure 3. (**a**) Relative (to *GAPDH*) mRNA expression determined by Q-RT-PCR of *ATP1A1* in proliferative (PRO, green) and senescence (SEN, red) A549 cells in basal conditions. (**b**) Relative (to *GAPDH*) mRNA expression determined by Q-RT-PCR of *ATP1A1* in proliferative (PRO, green) and senescence (SEN, red) A549 cells after transfection with control Scramble (Scr), or *ATP1A1* specific S1718 and S1720, siRNAs (left panel); and cell viability of these same cells treated with Digoxin (right panel). (**c**) Schematic diagram showing the increased levels of intracellular Ca2+ produced as a result of the inhibition of the Ca2+/Na+ Exchanger by the high concentrations of Na+ as a consequence of Digoxin inhibition of the Na+/K+ ATPase pump. (**d**) Intracellular calcium determination using fluorescent Calcium-6 probe of proliferative (PRO) or senescence (SEN) A549 cells treated or not with Digoxin, as indicated. n=32. (**e**) Relative cell viability (%) of proliferative (green) or senescence

(red) A549 cells treated with calcium chelator BAPTA-AM (BAP), Digoxin (Dig) or the combination of both (BAP+Dig). n=4. Data correspond to the average ± s.d. Statistical significance was assessed by the two-tailed Student´s t-test except (e) with Anova with Tuckey test: *** $p < 0.001$; * $p < 0.05$; *n.s.* > 0.05. Source data for these experiments are provided as a Source Data file.

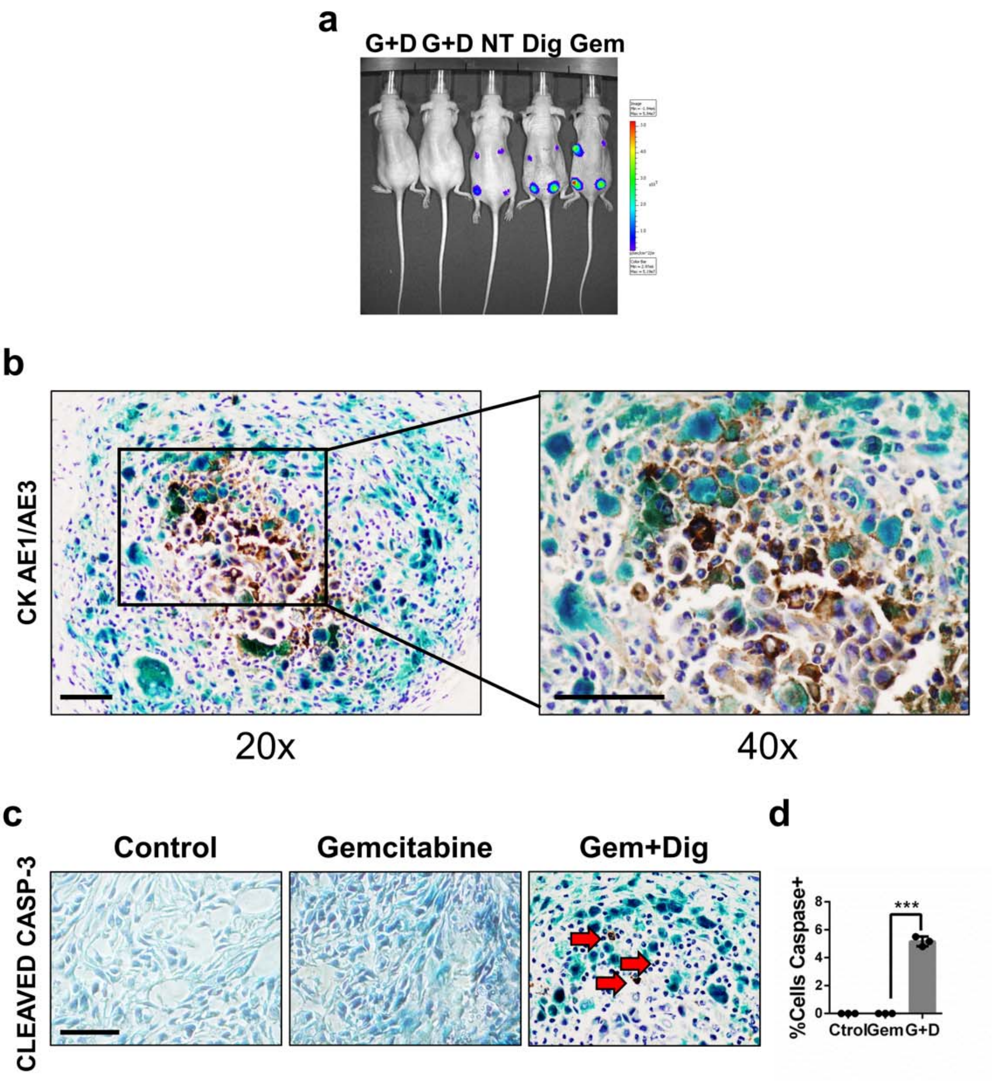

Supplementary Figure 4. (**a**) Representative image of nude mice under IVIS showing detectable tumors (luminescence signal) in the control (NT), Digoxin (Dig) or Gemcitabine (Gem) groups but not in the Gemcitabine plus Digoxin (G+D) group. (**b**) Representative pictures of immunohistochemical analysis of tumor marker CK AE1/AE3 in tissue sections from tumors derived from A549 subcutaneous injection in nude mice treated with Gemcitabine plus Digoxin (scale bar = 100 μm). (**c**) Representative pictures of immunohistochemical analysis of cleaved Caspase-3 (red arrows point to positive cells) in tissue sections from tumors derived from A549

subcutaneous injection in nude mice treated with Gemcitabine (middle panel), Gemcitabine plus Digoxin (right panel), or control (left panel) (scale bar = 100 μm). (**d**) Quantification of cleaved Caspase-3 positive cells. Data correspond to the average ± s.d. Statistical significance was assessed by the two-tailed Student´s t-test: *** $p < 0.001$. Source data for these experiments are provided as a Source Data file.

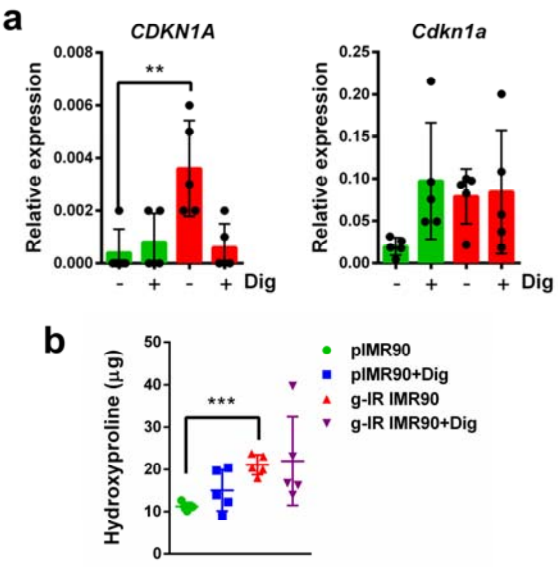

Supplementary Figure 5. (**a**) Relative (to *GAPDH*) mRNA expression of *CDKN1A* (left panel) and *Cdkn1a* (right panel) determined by Q-RT-PCR in mouse lung extracts after intratracheal administration of proliferative (green) or gamma-irradiated senescence (red) human IMR90 fibroblasts, treated (+) or not (-) with Digoxin (Dig). (**b**) Quantification of hydroxyproline in lungs of mice after intratracheal administration of proliferative (pIMR90) or gamma-irradiated senescence (g-IR IMR90) human IMR90 fibroblasts, treated (+Dig) or not with Digoxin. Data correspond to the average ± s.d. Statistical significance was assessed by the two-tailed Student´s t-test: *** $p < 0.001$, ** $p < 0.01$. Source data for these experiments are provided as a Source Data file.
