# Supplementary material for: Identification and characterization of Cardiac Glycosides as senolytic compounds
Source: Nat Commun. 2019 Oct 21;10:4731. doi: 10.1038/s41467-019-12888-x (PMC6803708; doi:10.1038/s41467-019-12888-x)
Supplement: Supplementary file 2 — Reporting Summary [file 41467_2019_12888_MOESM2_ESM.pdf]

nature research

Corresponding author(s): Manuel Collado

Last updated by author(s): Oct 4, 2019

Reporting Summary

Nature Research wishes to improve the reproducibility of the work that we publish. This form provides structure for consistency and transparency in reporting. For further information on Nature Research policies, see Authors & Referees and the Editorial Policy Checklist.

Statistics

For all statistical analyses, confirm that the following items are present in the figure legend, table legend, main text, or Methods section.

n/a	Confirmed	
☐	☒	The exact sample size (*n*) for each experimental group/condition, given as a discrete number and unit of measurement
☐	☒	A statement on whether measurements were taken from distinct samples or whether the same sample was measured repeatedly
☐	☒	The statistical test(s) used AND whether they are one- or two-sided<br>*Only common tests should be described solely by name; describe more complex techniques in the Methods section.*
☒	☐	A description of all covariates tested
☒	☐	A description of any assumptions or corrections, such as tests of normality and adjustment for multiple comparisons
☐	☒	A full description of the statistical parameters including central tendency (e.g. means) or other basic estimates (e.g. regression coefficient) AND variation (e.g. standard deviation) or associated estimates of uncertainty (e.g. confidence intervals)
☐	☒	For null hypothesis testing, the test statistic (e.g. *F*, *t*, *r*) with confidence intervals, effect sizes, degrees of freedom and *P* value noted<br>*Give P values as exact values whenever suitable.*
☒	☐	For Bayesian analysis, information on the choice of priors and Markov chain Monte Carlo settings
☒	☐	For hierarchical and complex designs, identification of the appropriate level for tests and full reporting of outcomes
☒	☐	Estimates of effect sizes (e.g. Cohen's *d*, Pearson's *r*), indicating how they were calculated

Our web collection on statistics for biologists contains articles on many of the points above.

Software and code

Policy information about availability of computer code

Data collection	N/A
Data analysis	N/A

For manuscripts utilizing custom algorithms or software that are central to the research but not yet described in published literature, software must be made available to editors/reviewers. We strongly encourage code deposition in a community repository (e.g. GitHub). See the Nature Research guidelines for submitting code & software for further information.

Data

Policy information about availability of data

All manuscripts must include a data availability statement. This statement should provide the following information, where applicable:

- Accession codes, unique identifiers, or web links for publicly available datasets
- A list of figures that have associated raw data
- A description of any restrictions on data availability

The data that support the findings of this study are available from the corresponding authors upon reasonable request. The source data underlying Figs 1c-f, 2a-c, 3b-i, 4b, c, e, 5c-f, 6c-f and Supplementary Figs 1a, b, c, e, f, g, 2, 3a, b, d, e, 4d, and 5a-b are provided as a Source Data file

Field-specific reporting

Please select the one below that is the best fit for your research. If you are not sure, read the appropriate sections before making your selection.

☒ Life sciences ☐ Behavioural & social sciences ☐ Ecological, evolutionary & environmental sciences

October 2018

For a reference copy of the document with all sections, see nature.com/documents/nr-reporting-summary-flat.pdf

Life sciences study design

All studies must disclose on these points even when the disclosure is negative.

Sample size	sample size was not pre-determined
Data exclusions	all analyzed samples were included for statistical analysis
Replication	all experimental procedures were performed at least three times with similar resuls
Randomization	mice were randomly assigned to treatment groups
Blinding	animals were analyzed by investifigators in a blind manner

Reporting for specific materials, systems and methods

We require information from authors about some types of materials, experimental systems and methods used in many studies. Here, indicate whether each material, system or method listed is relevant to your study. If you are not sure if a list item applies to your research, read the appropriate section before selecting a response.

Materials & experimental systems

n/a	Involved in the study	
☐	☒	Antibodies
☐	☒	Eukaryotic cell lines
☒	☐	Palaeontology
☐	☒	Animals and other organisms
☐	☒	Human research participants
☒	☐	Clinical data

Methods

n/a	Involved in the study	
☒	☐	ChIP-seq
☒	☐	Flow cytometry
☒	☐	MRI-based neuroimaging

Antibodies

Antibodies used	Prediluted flex monoclonal antibody to CKAE1AE3 (Dako-Agilent); Cleaved caspase 3 (clone 5A1E) (Cell Signalling Technologies); Ki67 (prediluted SP6, Master Diagnostica 0003110QD); p21Cip1 (HUGO-291 CNIO)
Validation	Commercial antibodies have been validated by producers but they were also tested routinely using tissue sections as positive and negative controls following standard procedures in the Pathology service of our Hospital in Santiago de Compostela. For the in-house Ab against p21Cip1, extensive validation was performed at the Histopathology service at CNIO, Madrid, Spain.

Eukaryotic cell lines

Policy information about cell lines

Cell line source(s)	ATCC
Authentication	none were authenticated
Mycoplasma contamination	All cell lines routinely tested negative for mycoplasma
Commonly misidentified lines (See ICLAC register)	None

Animals and other organisms

Policy information about studies involving animals; ARRIVE guidelines recommended for reporting animal research

Laboratory animals	Mus musculus, NMRInu/nu from Janvier Labs, female 8 weeks old
Wild animals	None
Field-collected samples	None
Ethics oversight	University of Santiago de Compostela Bioethics Committee (license number 15010/17/001); Vall d'Hebron following European,

Ethics oversight	Spanish and Catalan Regulations for Protection of Vertebrate Animals for Experimental and Scientific Purposes (Lab Animal Service Campus Vall d'Hebron (license number B9900062); and Ethical Committee for Animal Experimentation (CEEA) of the Scientific Park of Barcelona (PCB license number CEEA-19-029)

Note that full information on the approval of the study protocol must also be provided in the manuscript.

Human research participants

Policy information about studies involving human research participants

Population characteristics	N/A
Recruitment	N/A
Ethics oversight	Human breast tumors used to establish Patient-Derived Xenografts (PDXs) in this study were from biopsies or surgical resections at Vall d'Hebron University Hospital, Barcelona, and were obtained following institutional guidelines. The IRBs at Vall d'Hebron Hospital provided approval in accordance with the Declaration of Helsinki. Written informed consent was obtained from all patients who provided tissue

Note that full information on the approval of the study protocol must also be provided in the manuscript.
